# A Ship Grounding Over a Century Ago Left a Lasting Channel Among Corals

**DOI:** 10.1002/ece3.71857

**Published:** 2025-07-24

**Authors:** Thomas M. DeCarlo, Leticia Cavole, Gabriel Castro‐Falcón, Vinícius Ribau Mendes, Guilherme Ortigara Longo, Natan S. Pereira, Cristiano Mazur Chiessi

**Affiliations:** ^1^ Department of Earth and Environmental Sciences Tulane University New Orleans Louisiana USA; ^2^ School of Arts, Sciences and Humanities University of São Paulo São Paulo Brazil; ^3^ Institute of Marine Science Federal University of São Paulo Santos Brazil; ^4^ Marine Ecology Laboratory, Department of Oceanography and Limnology Federal University of Rio Grande Do Norte Natal Brazil; ^5^ PGQA, Department of Exact and Earth Science State University of Bahia Salvador Brazil

**Keywords:** aerial imagery, coral reef, ship grounding, shipwreck

## Abstract

Among disturbance events to coral reef ecosystems, ship groundings can be among the most acute due to the physical damage they cause to coral reef habitats. Following ship groundings, monitoring studies show that some reefs recover whereas others retain changes in coral community structure for at least a decade. Thus, the recovery timescales following groundings are variable, but the general paradigm is that reef communities will begin on a trajectory toward recovery to the pre‐disturbance state. Here, we report several lines of evidence of a 100+ year old ship grounding in northeastern Brazil. Strikingly, the ship grounding led to a semi‐permanent sand channel in the reef that has not substantially trended toward recovery. Our observations support the notion that acute disturbance on coral reefs can cause structural changes that may never return to the pre‐disturbance conditions.

## Graphical abstract

Ship groundings damage coral ecosystems due to the physical abrasion or shattering of corals, but also lead to changes in community structure and increases in coral diseases. In northeastern Brazil, the state of Rio Grande do Norte has a notable history of shipwrecks and maritime incidents due to the combination of strong trade winds, complex currents, and shallow coral reefs. We observed what appears to be a sand channel cut through a coral community by a ship grounding more than 100 years ago in the parrachos of Rio do Fogo.

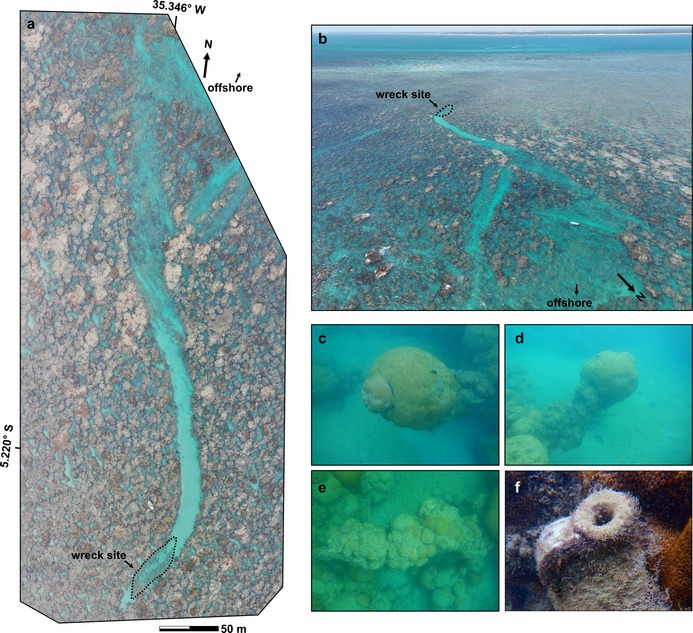

Coral reefs are notorious navigational hazards where ships run aground. Ship groundings damage coral ecosystems due to the physical abrasion or shattering of corals and lead to changes in community structure and increases in coral diseases (Victoria‐Salazar et al. 2017; Raymundo et al. 2018). Due to the common occurrence of groundings, even in an era of modern global positioning systems, several studies have tracked long‐term coral community recovery and assessed restoration efforts (Precht et al. 2001; Cameron et al. 2016; Victoria‐Salazar et al. 2017; Wever 2022; Morris et al. 2024). In some cases, coral community structure recovered to be indistinguishable from adjacent undisturbed sites within 10 years (Precht et al. 2001; Morris et al. 2024), whereas in other cases there was not substantial recovery of the coral community over 10 or more years despite management and/or active restoration (e.g., outplanting) efforts (Precht et al. 2001; Victoria‐Salazar et al. 2017; Morris et al. 2024). Thus, even longer‐term studies may be needed to identify the range of potential outcomes for coral communities impacted by ship groundings.

In northeastern Brazil, the state of Rio Grande do Norte harbors a significant shallow reef system that has been associated with a notable history of shipwrecks and maritime incidents due to the combination of strong trade winds, complex currents, and shallow coral reefs (Figure 1). Historical records on the biodiversity of these reefs date back to the 1970s, from exploratory expeditions by the French naturalist Jacques Laborel (Longo 2019). Laborel described a vast reef bank located 5–7 km offshore, highlighting the Cioba reefs (Touros municipality), Fogo reefs (Rio do Fogo municipality), and Maracajaú reefs (Maxaranguape municipality). The offshore reef banks, such as those we studied in Rio do Fogo, consist of pinnacle formations at depths of 2–6 m, regionally known as “parrachos”. The spatial arrangement and elongation of most coastal coral reefs in this region suggest they formed over sandstone outcrops that are themselves ancient coastlines that have been submerged and consolidated due to sea level fluctuations during the Holocene (Testa and Bosence 1999). The structure of the *parrachos* is mainly composed of calcareous algae, vermetid gastropods, and corals, with a vertical thickness of 2–4 m (Longo 2019). In the 1970s, the coral *Siderastrea stellata* composed around 80% of the framework through large rounded colonies, alongside *Porites astreoides* and the hydrocoral *Millepora alcicornis* , which formed crowns atop the reef. This scenario is still observed today, but with much less *M. alcicornis* , which have suffered up to 75% mortality after consecutive thermal stress events (Rodrigues et al. 2025). Laborel's description also notes that the reef was covered by a carpet of algae—particularly the green alga *Caulerpa racemosa* —and the zoanthid *Palythoa caribaeorum*. Currently, the Rio do Fogo *parrachos* show moderate spatial variation in benthic composition, mainly composed of algal turfs, brown algae (Dictyotaceae), *P. caribaeorum*, and *S. stellata* (Roos et al. 2019). These reefs also host other coral species (including *Agaricia humilis, Favia gravida, Mussismilia harttii, Porites branneri*, and *Montastraea cavernosa* ) and hydrocorals ( *Millepora braziliensis* ). Despite these ecological studies in the area, none of them have investigated or described the impact of shipwrecks on these reefs.

**FIGURE 1:**
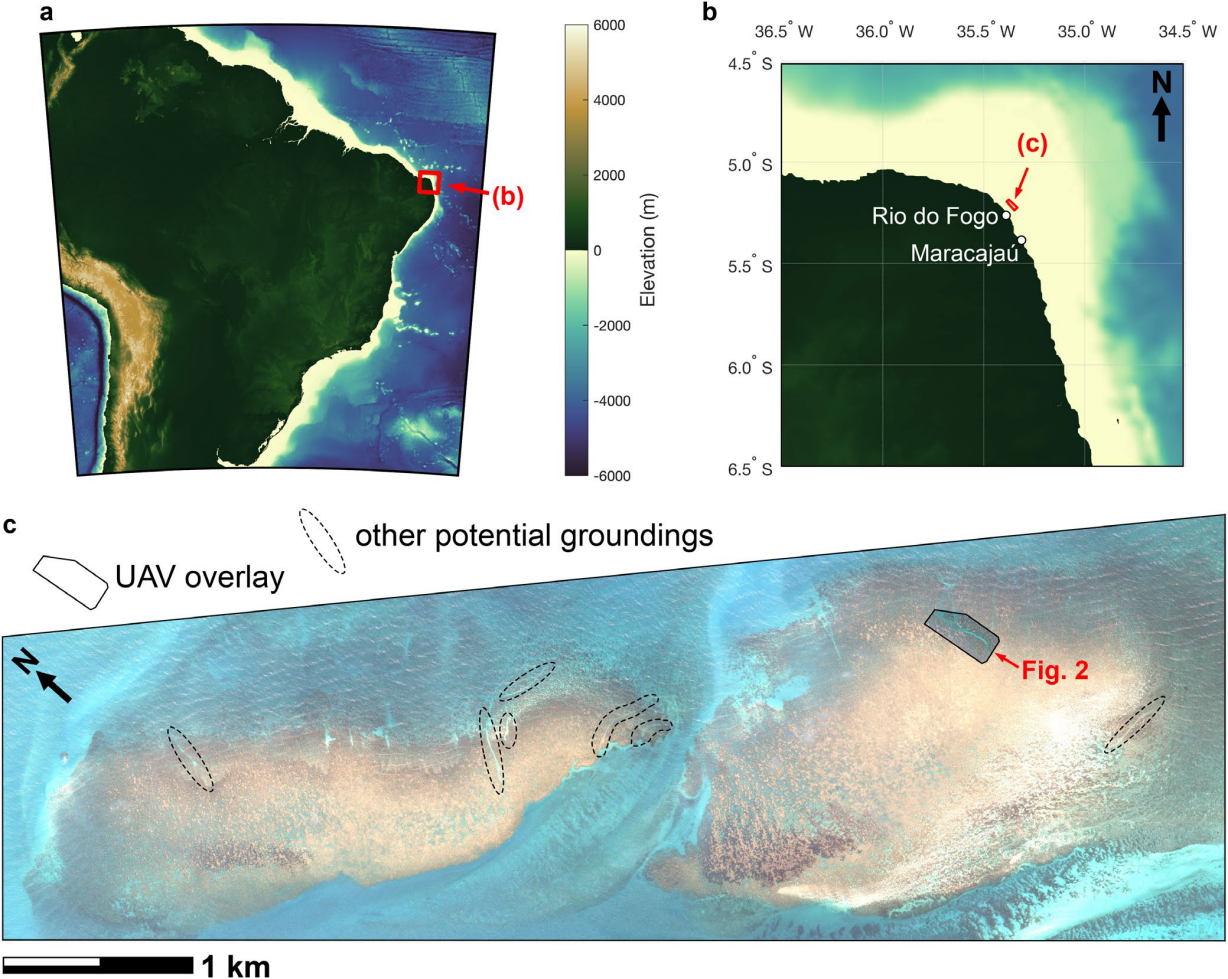
Regional map and high‐resolution satellite image of the Rio do Fogo parrachos. Bathymetry maps (a, b) were made using ETOPO1 data (Amante and Eakins 2009). Satellite imagery in panel (c) is from Pleiades satellite system, 50 cm resolution, taken 17 February, 2022. The solid black outline in panel (c) shows the UAV photomosaic from Figure 2 superimposed on the satellite imagery and the dashed black outlines show potential other sand channels created by ship groundings.

According to the Brazilian Shipwrecks database (Sistema de Informações de Naufrágios (SINAU), n.d.), 118 shipwrecks have been documented in the region since the 17th century. Historical records reveal that large ships frequently ran aground on the shallow reefs of Rio do Fogo, particularly in the 19th century. In 1840, the English ship Orion—measuring between 40 and 90 m in length and weighing about 200 t—sank while transporting coffee from Brazil to England. A few years later, another English vessel, the N.D. Callie, carrying timber, also sank in the same area and, in 1885, the Dutch ship Stella transporting salt from Brazil to the Netherlands met a similar fate. Multiple shipwrecks were also recorded at an adjacent reef area, Maracajaú, mostly of cargo ships in the 19th century. While it remains unclear which ship caused specific grounding scars, these historical accounts provide strong evidence that shipwrecks were a common occurrence on the *parrachos*. Based on a combination of in‐water visual inspections and aerial photographs, we observed what appears to be a sand channel cut through the coral community by a ship grounding more than 100 years ago in the *parrachos* of Rio do Fogo (5.22°S, 35.35°W). Several lines of evidence led us to this conclusion.

At the inshore terminus of the channel are metal remains of the skeleton of a ship's hull, covered by *S. stellata* coral colonies. The outline of the hull is visible in aerial photographs taken on 13 March 2025 from an unmanned aerial vehicle (UAV; a DJI Mavic 3 Pro model), labeled as “wreck site” in Figure 2a. The aerial photograph in Figure 2a is a photomosaic that was compiled from 37 individual photographs, georeferenced, and formed into an orthomosaic using Pix4Dmatic software. Underwater, metal (likely iron) pieces in a concave‐up shape stretch from one side of the channel to the other, now covered by massive *S. stellata* colonies (Figure 2c–e). We also found a ceramic jug nearby that coral had mostly encrusted over (Figure 2f). The age of the grounding, and the name of the ship, is not precisely known, but several indicators suggest it is at least 100 years old. First, the wreck appeared to be a wood‐iron composite ship, which were constructed for trade during the second half of the 19th century (Rodriguez 2012). These construction characteristics align well with those of both English ships, referred as barks in the historical records, and the Dutch cargo sail ship that sank in the area in the late 19th century. While the time of construction is necessarily earlier than the grounding, the construction mode of these ships was largely outdated by the early 20th century. Second, the *S. stellata* colonies growing on the hull were approximately 30–40 cm in height. Previous analyses of *S. stellata* cores nearby at Maracajaú found growth rates of 3.8 ± 0.7 mm per year (Pereira et al. 2022), but preliminary measurements in cores collected adjacent to the Rio do Fogo shipwreck show slower growth of 2.7 ± 0.6 (1 SD) mm per year (L. Cavole, unpublished data), giving an estimated age of ~90–150 years (plus an unknown duration between the grounding and the settlement of the colonies still living today). Finally, local fishers were familiar with this shipwreck site and the story of the wreck had been passed down orally across at least three generations. They told us the wreck was over 100 years old and that local fishers had gathered the wood from the hull after the wreck (pers. comm. Janildo and Manoel Gomes de Santana), consistent with our interpretations above. There are also historical records at the “Historical and Geographical Institute of Rio Grande do Norte” describing how the local population helped save victims and a legal document determining that the coffee from the English ship that sank should be divided among those that helped.

**FIGURE 2:**
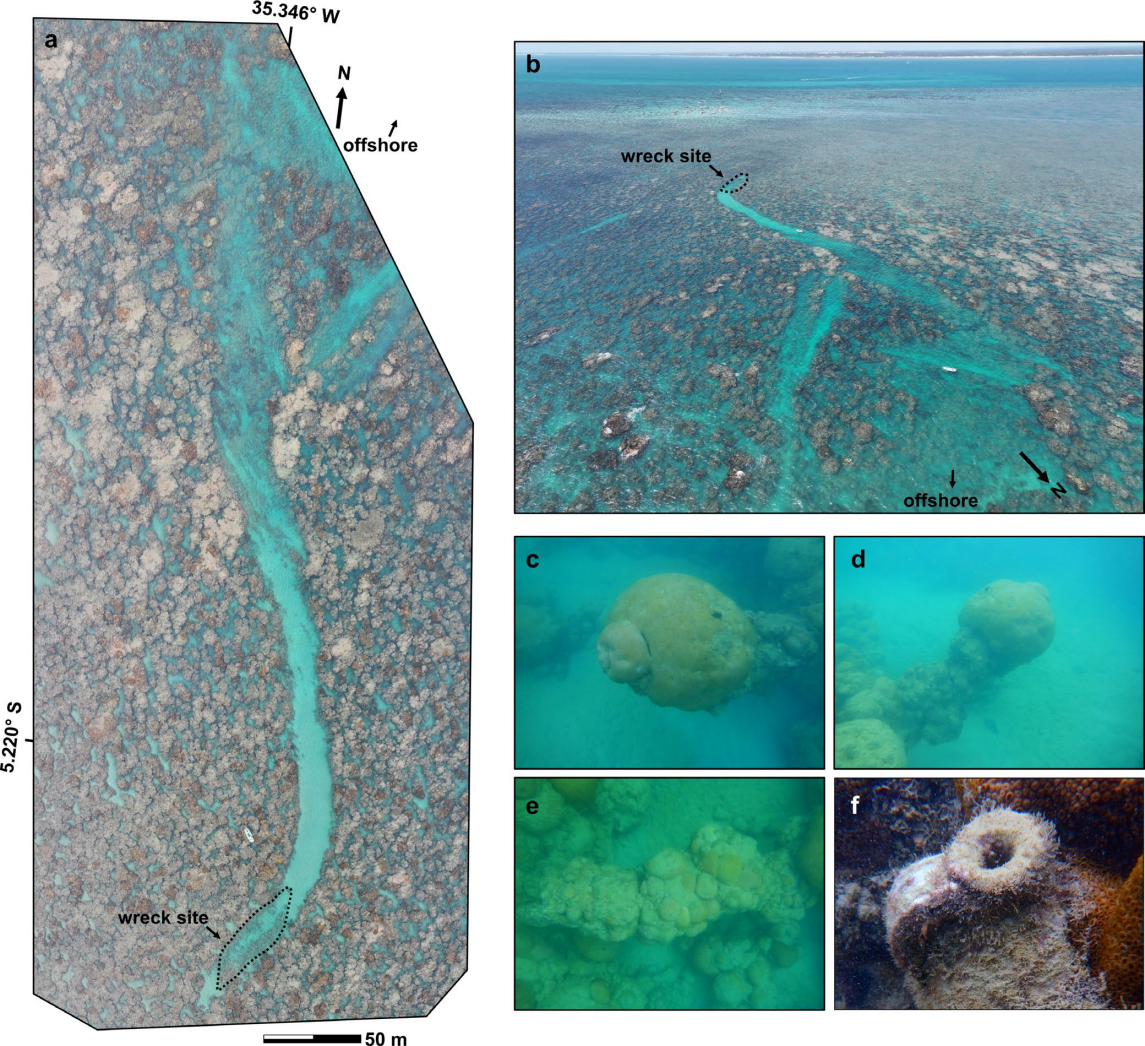
Aerial and underwater images of a sand channel carved by a ship grounding in Rio do Fogo parrachos, Brazil. (a) Aerial photomosaic of the channel including the wreck site, constructed with photographs taken 13 March 2025. (b) Single aerial photograph taken 12 March 2025 from offshore and looking toward the channel and wreck. (c–e) Underwater photographs of corals growing on the remains of the ship hull. (f) Ceramic jug nearby the wreck. Photo credit: Thomas M. DeCarlo (panels a, b) and Gabriel Castro‐Falcón (panels c–f).

Additional evidence supports our conclusions that the channel visible in Figure 2a,b was cut by the ship grounding. The channel runs from the offshore extent of the shallow coral community for several hundred meters across the shallow *parrachos* before abruptly ending, which is unlike natural reef channels that typically connect lagoons to offshore or channels within spur and groove topography that begin on the outer reef slope (Duce et al. 2016). Further, the shipwreck is located exactly at the terminus of the channel, and the channel width (~15 m) appears to closely match the width of the vessel (based on underwater surveys of shipwreck features subsequently measured in the aerial photomosaic), which strongly supports our conclusion that the channel was created by the grounding. We suggest that it would have been nearly impossible for the vessel to navigate such a pre‐existing channel, including a bend in its path, and reach the end of the channel. Rather, we postulate that the grounding led to a self‐sustaining sand channel. This could have occurred if the vessel sufficiently gouged and/or broke the framework to destabilize material along the grounding path, not necessarily that the vessel plowed the entire channel clear of coral framework. If this created a preferential pathway for sand generated within the coral community to be exported offshore along the bottom of the channel, similar to the direction of material transport in spur and groove topography (Rogers et al. 2013), the channel could have matured following the ship grounding. Now, the loose sand in the channel prevents coral settlement, and the abrasion of the coral framework by moving sand on the sides of the channel likely sustains the channel's shape. We suggest these processes have been ongoing for more than a century, probably since the late 19th century, extending the duration of observed ship grounding impacts to coral communities and providing an example of a potentially permanent alteration to physical coral framework and sediment transport following a ship grounding. An implication of these findings is that acute disturbance events to coral habitat are not necessarily followed by recovery trajectories pointing back to the original state, but rather that they can cause permanent physical changes to the reef framework and consequently to reef community dynamics (Hatcher 1984; Work et al. 2008; Dudgeon et al. 2010).

High‐resolution satellite images of the Rio do Fogo *parrachos* show what appear to be several other channels potentially cut by ship groundings (Figure 1). The ship grounding we describe is not an isolated or rare occurrence, as indicated by the historical records of multiple shipwrecks in this area, but rather one clear example where the impacts of the grounding can be deduced. Given the apparently high occurrence in Rio do Fogo, ship groundings have likely transformed the area's coral ecosystems, and ship groundings could be important habitat and ecological drivers potentially across larger geographic regions if they occur at sufficient frequency. Thus, our findings highlight the importance of active restoration efforts following ship groundings on coral reefs (Wever 2022; Leung et al. 2024). Further, while the primary limitation for coral re‐population in these channels created by ship groundings is likely to be sand scour and substrate instability (Kenyon et al. 2023), recent heat‐induced coral bleaching events in the region (Rodrigues et al. 2025) are likely only decreasing the capacity of corals to repopulate the ship‐grounding channels.

## Author Contributions

**Thomas M. DeCarlo:** conceptualization (equal), formal analysis (equal), investigation (equal), resources (equal), visualization (equal), writing – original draft (equal). **Leticia Cavole:** funding acquisition (equal), investigation (equal), writing – review and editing (equal). **Gabriel Castro‐Falcón:** formal analysis (equal), investigation (equal), writing – review and editing (equal). **Vinícius Ribau Mendes:** formal analysis (equal), investigation (equal), writing – review and editing (equal). **Guilherme Ortigara Longo:** conceptualization (equal), writing – review and editing (equal). **Natan S. Pereira:** funding acquisition (equal), resources (equal), writing – review and editing (equal). **Cristiano Mazur Chiessi:** conceptualization (equal), funding acquisition (equal), writing – review and editing (equal).

## Conflicts of Interest

The authors declare no conflicts of interest.

## Data Availability

The authors confirm that all data are available in the manuscript as imagery data. Full‐resolution images are available on Zenodo at https://doi.org/10.5281/zenodo.15686164.
